# On the effectiveness of network metrics on key class prediction: An empirical study

**DOI:** 10.1371/journal.pone.0334408

**Published:** 2025-10-10

**Authors:** Shiyuan Zhou, Wei Wu, Jiale Wang, Hongbing Liu, Chenxiang Yuan

**Affiliations:** 1 School of Information Engineering, Jiaxing Nanhu University, Jiaxing, China; 2 School of Computer Science and Technology, Zhejiang Gongshang University, Hangzhou, China; Lodz University of Technology: Politechnika Lodzka, POLAND

## Abstract

Key classes are the most important classes in a software system, which provide an excellent foundation for developers—especially those new to the field—to understand unfamiliar software systems. In the past decade, several key class prediction (KCP) approaches have been proposed. They used *design metrics* extracted from source code and *unweighted network metrics* computed on class coupling networks as *features* and built machine-learning models to predict whether a class is a key class or not. However, previous studies mainly focused on improving the performance of KCP models in the *within-project* (i.e., KCP in the same project) context, and the *network metrics* they used are *unweighted* and *inaccurate*, as they are computed on unweighted and incomplete class coupling networks. These limitations lead to a lack of thorough evaluation of the effectiveness of *network metrics* for KCP, especially in the *cross-project* (KCP across diverse projects) context, which in turn results in uncertainty about how to choose suitable metrics as *features* when building KCP models. To fill this gap, in this paper, we thoroughly evaluate the effectiveness of network metrics for KCP. Specifically, we build weighted and more complete class coupling networks for software, and introduce a set of weighted network metrics to characterize class complexity. Then, we build different KCP models using the *Random Forest* learner and the *Naive Bayes* model for two KCP contexts (i.e., *within-project* and *cross-project*), respectively, with *design metrics*, *unweighted/weighted network metrics*, and their combinations being *features*. Finally, through an empirical study on 18 open-source Java projects, we thoroughly investigate the relative effectiveness of *network metrics* over *design metrics* across the two KCP contexts. Our results suggest that when building KCP models, to achieve better performance, researchers and practitioners should consider using unweighted (or weighted) *network metrics* alone or along with *design metrics* in the *within-project* KCP context, using *design metrics* alone or along with unweighted (or weighted) *network metrics* in the *cross-project* KCP context, and using unweighted (or weighted) *network metrics* along with *design metrics* across the two KCP contexts.

## 1 Introduction

Software comprehension is recognized as a daunting and time-consuming task, which occupies more than 50% of the time allocated to maintain a specific software project [1,2]. To ease the process of understanding software projects, developers usually refer to their design documentations [2]. However, as software evolves, design documentations are seldom up-to-date. For legacy software, documentations might even be unavailable [2]. Reverse engineered class diagrams can be used as a replacement. However, they might contain much details when the size of the software is large, and thus provide little benefit to software comprehension [2–4]. It would be helpful if some effective techniques or tools could be developed to condense class diagrams so as to provide better support for software comprehension [2,3,5].

To condense class diagrams, one possible way is to identify the most important classes (aka *key classes*) and discard unimportant ones [2,3,5]. In the literature, *key classes* are usually described as “*classes managing a large number of other classes*", “*classes using a large number of other classes to implement their functionality*", and “*classes tightly coupled with other parts of the system*" [6–8]. If the condensed diagram composed of *key classes* is closer to the design documentation, then it will benefit the software comprehension task [5,9].

In the past decade, several key class prediction (KCP) approaches have been proposed to predict whether a class is a key class or not [3,5,10]. They used *design metrics* (denoted as DMs hereafter) alone [3] or along with *unweighted network metrics* (denoted as NMs hereafter) [5,10] as input *features* to classification algorithms to build prediction models. Actually, in Refs. [5] and [10], authors referred to their metrics derived from unweighted class coupling networks as *network metrics*. In this study, we propose a weighted variant of their *network metrics*. Thus, to differentiate between the two distinct sets of *network metrics*, we refer to their *network metrics* as *unweighted network metrics* and ours as *weighted network metrics*. They both are referred to as *network metrics*. Ref [3] found that Random Forest trained on DMs can achieve promising performance on KCP. Ref [5] found that DMs along with NMs outperform DMs alone on KCP with a 9.1% improvement of the *AUC* (area under the receiver operating characteristic curve) score. Ref [10] improved Thung et al.’s work by training an ensemble classifier on both DMs and NMs. Although some advances have been made in KCP, there are still some unsolved problems: i) The class coupling networks they built to represent class-level software structures (i.e., classes and their couplings), and compute NMs are not very accurate, discarding many important couplings (e.g., method calls) between classes, as well as the weight on edges. Such an inaccurate (or incomplete) representation will make the obtained NMs values incorrect and even lead previous studies to draw erroneous conclusions. ii) The NMs used in previous studies only fits in with unweighted networks. They cannot capture the coupling strength between classes and thus cannot reflect the actual complexity of classes, which might affect the performance of KCP models built on them. iii) The previous KCP models are mainly proposed for the *within-project* context, i.e., the training data and testing data all come from the same version of a specific project. There are no previous studies targeting the *cross-project* KCP, i.e., to predict key classes in the current project (testing data), the training data are from other different projects. iv) The previous KCP studies used the same set of 9 open source Java projects as research subjects. The scale is small, which may affect the generalization of the obtained conclusions. v) The previous studies only built KCP models on DMs alone or along with NMs, with no attention paid to building KCP models on NMs alone. These limitations lead to a lack of thorough evaluation of the relative effectiveness of *network metrics* (i.e., NMs and weighted NMs) over DMs for KCP, which in turn results in uncertainty about how to choose suitable metrics as *features* when building KCP models.

To tackle the above problem, we set out to investigate the effectiveness of *network metrics* for KCP with the aim to find out i) the relative effectiveness of *network metrics* over DMs, and ii) which metric suite or metric suite combination performs best. First, we build a weighted directed network, ${\text{CCN}}_{\text{WD}}$ (Weighted Directed Class Coupling Network), to represent classes and their couplings in software. Second, we introduce a set of weighted network metrics (denoted as NMs_*w*_ hereafter) to characterize the complexity of classes in a ${\text{CCN}}_{\text{WD}}$. NMs_*w*_ is a weighted variant of NMs. We also compute DMs and NMs for all the classes in the project. Third, we use DMs, NMs, and NMs_*w*_ alone or their combinations (i.e., DMs+NMs, DMs+NMs_*w*_, NMs+NMs_*w*_, and DMs+NMs+NMs_*w*_) as input *features* for two classification algorithms (i.e., *Random Forest* and *Naive Bayes*) to build KCP models for two contexts (i.e., *within-project* and *cross-project*). Finally, by comparing the performance of different models on a set of 18 open-source Java projects collected from the KCP literature, we can reveal the relative effectiveness of *network metrics* over DMs, and find the best metric suite or metric suite combination for KCP in different contexts.

The main contributions of our study are:

- We improve the quality of NMs data already used in the previous studies. Specifically, we introduce a weighted directed network (i.e., ${\text{CCN}}_{\text{WD}}$), which can capture more coupling types as well as the coupling strength between classes. Based on ${\text{CCN}}_{\text{WD}}$s, we recompute the value of NMs and reexamine its relative effectiveness over DMs on KCP.
- We introduce a weighted variant of NMs—NMs_*w*_, which is helpful to capture the coupling strength on the complexity as well as the importance of classes. To our knowledge, it is the first study to examine the effectiveness of NMs_*w*_ on KCP.
- *Cross-project* is a new KCP context, which has never been explored in the KCP literature. In this study, we examine the relative effectiveness of *network metrics* (i.e., NMs and NMs_*w*_) over DMs across two KCP contexts (i.e., *within-project* and *cross-project*).
- We investigate the relative effectiveness of *network metrics* over DMs separately by training KCP models solely on one of the network metric suites — that is, “NMs vs. DMs" and “NMs_*w*_ vs. DMs". We also investigate the situation in which network metrics are combined with DMs—that is, “(NMs+DMs) vs. DMs", “(NMs_*w*_+DMs) vs. DMs", and “(NMs+NMs_*w*_+DMs) vs. DMs".
- Our experiments are performed on 18 open-source Java projects. To our knowledge, it is the largest data set so far, as the previous KCP studies only used 9 out of the 18 projects. Such a large data set can increase the confidence of the obtained conclusions.

We structure our work by addressing the following two research questions:

- RQ1: *Do network metrics outperform design metrics in the within-project KCP context?*
  In the within-project KCP context, models trained on network metrics (i.e., NMs and NMs_*w*_) alone or along with design metrics (i.e., DMs) outperform models trained on DMs alone on KCP. Furthermore, models trained on NMs_*w*_ are superior to both models trained on NMs and models trained on NMs+DMs. We find that such a result may come from the fact that NMs_*w*_ can discriminate key classes better than NMs, and NMs can discriminate key classes better than DMs. Thus, we suggest that researchers and practitioners should consider using network metrics alone or along with design metrics to train KCP models in the within-project context.
- RQ2: *Do network metrics outperform design metrics in the cross-project KCP context?*
  In the cross-project KCP context, models trained on design metrics (i.e., DMs) outperform models trained on network metrics (i.e., NMs and NMs_*w*_). In addition, models trained on network metrics along with design metrics do better than models trained on network metrics alone. We find that such a result may come from the fact that design metrics are less project-specific and thus are more suitable to build cross-project KCP models. Thus, we suggest that researchers and practitioners should consider using design metrics alone or along with network metrics to train KCP models in the cross-project context.

*Paper Organization.* Sect 2 describes our study design, including the subject systems, the software metric suites used, the KCP contexts considered, and the experiment setup. Sect 3 presents and analyzes the experimental results. Sect 4 and Sect 5 present the threats to the validity of our work and implications for software engineering, respectively. Sect 6 presents the related work. Finally, we give conclusions and discuss our future work in Sect 7.

## 2 Study design

**Fig 1:**
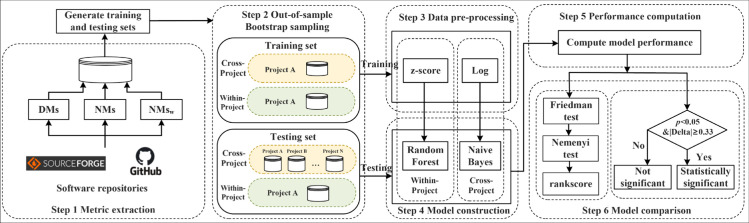
An overview of our study design.

In this section, we describe the subject systems used in the experiments, and the main steps (see Fig 1) used to build and evaluate different KCP models so as to address our research questions raised in Sect 1.

### 2.1 Subject systems

We collected 18 open-source Java projects (see Table 1) as our research subjects, which is a union of the subject systems that can be found in the KCP literature [3,5,10] and other unsupervised key class identification studies (Unsupervised key class identification approaches propose some metrics to measure class importance so as to identify key classes. They do not depend on any machine-learning algorithms) [7,8,11–15]. To our knowledge, it is the largest data set so far.

**Table 1:** Descriptions of the subject systems.

Systems	Version	Directory	*KLOC*	*#P*	*#C*	*#M*	*#A*	*#KC*	*IR*	Website
ArgoUML	0.9.5	src	74.334	67	846	6,178	2,851	12	mygray1.4%	http://argouml.sourceforge.net
Mars	3.06	src	132.589	95	1,085(32)	11,105	5,738	29	mygray2.6%	http://mars-sim.sourceforge.net/
javaclient	2	src	12.053	39	215	1,479	1,009	57	26.5%	http://java-player.sourceforge.net/
JGAP	3.6.3	src	29.043	27	411(5)	3,186	1,271	18	4.3%	http://sourceforge.net/projects/jgap/
Neuroph	2.2	src	13.657	24	172(4)	1,063	916	24	13.6%	http://neuroph.sourceforge.net/
JPMC	20020123	src	9.283	15	147	926	353	24	16.3%	http://jpmc.sourceforge.net/
wro4j	1.6.3	src	33.736	99	567(9)	3,256	1,274	12	mygray2.1%	http://code.google.com/p/wro4j/
xuml-compiler	0.4.8	all	25.502	58	388(3)	2,919	1,544	37	9.5%	http://code.google.com/p/xuml-compiler/
Maze	1	src	8.881	6	63(6)	563	284	27	39.1%	http://code.google.com/p/maze-solver/
Ant	1.6.1	src/main	81.515	67	900	7,691	4,167	10	mygray1.1%^2^	http://ant.apache.org/
jEdit	5.1.0	src	112.492	41	1,082(9)	7,601	4,085	7	mygray0.6%	http://www.jedit.org/
jHotDraw	6.0b.1	src	28.330	30	544	5,205	865	9	mygray1.7%	http://sourceforge.net/projects/jhotdraw/
jMeter	2.0.1	src/core	22.701	42	260	2,000	834	14	5.4%	http://jmeter.apache.org/
GWT Portlets	0.9.5beta	src	8.501	10	131	1,145	424	27	20.6%	http://code.google.com/p/gwtportlets/
tomcat	7.0.10	src	187.060	136	1,900(24)	16,631	7,950	28	mygray1.5%	http://tomcat.apache.org/
log4j	2.3	src	69.456	91	1,212(25)	6,689	3,101	9	mygray0.7%	https://logging.apache.org/log4j/
PDFBox	2.0.7	all^1^	135.514	109	1,285(33)	10,482	4,792	12	mygray0.9%	https://pdfbox.apache.org/
Xerces	2.11.0	all	133.663	71	1,256	10,481	5,819	6	mygray0.4%	https://xerces.apache.org/

^1^*all* means all directories in the source code distribution of the system. ^2^ The cells whose *IR* value ≤3 are marked in gray.

An overview of the subject systems is shown in Table 1, where *Systems* lists the software name, *Version* is the analyzed software version, *Directory* presents analyzed code directories in the source code distribution, and *KLOC* shows the number of source code lines in thousands in the analyzed code directories. Furthermore, *#P*, *#C*, *#M*, and *#A* contain the number (#) of packages, classes, methods, and attributes, respectively. *#KC* gives the # of true key classes in each system, IR=$\frac{\#KC}{\#C}$ (i.e., *imbalance rate* [16]) shows the percentage of true key classes, and *Website* gives the URLs to download each system. In this work, *KLOC* ignores both the # of comment lines and # of blank lines, and *#P* excludes the # of imported packages. *#C* is displayed in the form of “X(Y)", where *X* is the sum of # of classes, inner classes, and interfaces, and *Y* is the number of enum types.

To our knowledge, all previous KCP studies [3,5,10] only experimented on the first nine projects in Table 1, and they built the *ground truth* for each system by comparing the *reverse-engineered class diagram* (RED) and the *forward designed class diagram* (FDD). Specifically, if a class exists in both diagrams, then it is labeled as a *key* class. If a class exists in the RED, but not in the FDD, then it is labeled as a *non-key* class. However, we found that the REDs built in the literature are inaccurate. For example, as reported in Refs. [3,5] and [10], there are only 84 and 87 classes in xUML and wro4j, respectively. But we found that the two systems actually have 388 and 567 classes, respectively (see Table 1).

Due to the low quality of the data set used in the previous KCP studies, we do not use the *ground truth* they built. For our study, we adopted a more rigorous approach by employing the widely acknowledged *ground truth* used in key class identification literature [7,8,11–15,17]. In these references, researchers have proposed various unsupervised approaches to identify key classes. Specifically, these methodologies involve extracting key classes from the design documentations via phrases like *architectural overview* and *core of the system* in free texts or pruned diagrams [7,15,17]. In this work, the *ground truth* for each subject system was directly collected from these previous studies.

Note that the last nine projects in Table 1 have never been used in any previous KCP studies. We use them here with the aim to ensure the generalization of our obtained conclusions, as they are nontrivial projects, exhibit heterogeneity in their sizes, and come from different application domains. Interested readers can refer to Sect 1 of our online Appendix (https://github.com/SEGroupZJGSU/KCP/tree/main/Appendix) for a brief description of each system.

### 2.2 Software metric suites

Software metrics are usually used to capture different aspects of complexity enclosed in a software system. They are widely used in software engineering fields, such as defect prediction [18], change prediction, and research related to key class problems, which is relevant to this paper. In this section, we first briefly describe two metric suites that have been used in previous KCP studies: DMs and NMs. Then, we detail our new set of network metrics, NMs_*w*_, which is a weighted variant of NMs. We also introduce four metric suites, which are combinations of DMs, NMs, and NMs_*w*_.

#### 2.2.1 Design metric suite.

Ref [3] built a design metric suite, which consists of 11 metrics: five size-related metrics and six coupling-related metrics (see Table 2). The size-related metrics focus on measuring the size of a class from different angles and facets, and the coupling-related metrics are designed to measure the frequencies of couplings that a class has with other classes. The 11 design metrics are listed in Table 2, and their detailed descriptions can be found in Sect 2 of our online Appendix (https://github.com/SEGroupZJGSU/KCP/tree/main/Appendix).

**Table 2:** Software metrics used in our study.

Metric families	Types	Metrics
Design Metrics (DMs) [3]	Size	*NumAttr*, *NumOps*, *NumPubOps*, *Setters*, *Getters*
	Coupling	*Dep_Out*, *Dep_In*, *EC_Attr*, *IC_Attr*, *EC_Par*, *IC_Par*
Unweighted Network Metrics (NMs) [5,10]	Network	*baryC*, *betweenC*, *closeC*, *EigenC*, *hub*, *auth*, *PR*
Weighted Network Metrics (NMs_*w*_)	Network	*baryC*_*w*_, *betweenC*_*w*_, *closeC*_*w*_, *EigenC*_*w*_, *hub*_*w*_, *auth*_*w*_, *PR*_*w*_

Ref [3] used two publicly available tools, MagicDraw 16.5 (https://www.3ds.com/products-services/catia/products/no-magic/magicdraw/) and SDMetrics V2.5 (https://www.sdmetrics.com/), to compute the 11 metrics. MagicDraw is a software modeling tool, which can construct REDs from the source code of a software project. SDMetrics is an object-oriented (OO) design quality measurement tool, which can analyze REDs and compute the 11 metrics for classes. For our study, we also used the two tools to compute the 11 metrics.

#### 2.2.2 Unweighted network metric suite.

Ref [5] introduced an unweighted (the term “unweighted" is used to signify that these metrics are designed for unweighted networks) network metric suite (i.e., NMs) for KCP, which is composed of 7 network metrics (see Table 2) for unweighted networks. These metrics are mainly used to measure the *centrality* (or *importance*) of nodes in the whole network. It is a *macro*, *overall*, or *global* perspective, which is very different from the *micro* or *local* perspective that design metrics used. The *macro* perspective focuses on the network as a whole. However, the *local* perspective only focuses on the class itself or its one-step neighbors. Table 2 shows the 7 unweighted network metrics. Interested readers can refer to Sect 3 of our online Appendix (https://github.com/SEGroupZJGSU/KCP/tree/main/Appendix) for a detailed description.

To compute NMs, Ref [5] represented software as a *class network*. However, as pointed out in Sect 1, the *class networks* that [5] and [10] used to compute NMs are inaccurate, making the obtained metric values inaccurate. Thus, for our study, we use an improved *class network*, ${\text{CCN}}_{\text{WD}}$ (Weighted Directed Class Coupling Network), to represent classes (if not mentioned explicitly, the term *class* designates classes, interfaces, and enum types hereafter) and their couplings in software projects. ${\text{CCN}}_{\text{WD}}$ is first proposed in previous work [17,19].

${\text{CCN}}_{\text{WD}}$ is actually a weighted directed network (or graph), which can be formally defined as

$${\text{CCN}}_{\text{WD}}=(N,L,W)$$ (1)

where *N* is a *node* set, denoting all the classes in a project; L={⟨u,v⟩|u,v∈N∧u≠v∧w⟨u,v⟩>0} is a *link* set, denoting all the couplings that exist between any pairs of classes; and W={w⟨u,v⟩|⟨u,v⟩∈L} is a *weight* set, denoting the weights associated with links.

In ${\text{CCN}}_{\text{WD}}$s, we do not allow ≥2 links from *u* to v (u,v∈N), i.e., we only keep one if ≥2 couplings exist. The weight associated with the link ⟨u,v⟩∈L, w⟨u,v⟩, is computed by

$$w\left\langle u,v\right\rangle =\sum_{𝑐\in CS}(frq_{c}\times s_{c})$$ (2)

where CS is a *coupling* set, containing all the couplings from *u* to v, *c* is a specific *coupling* type, $frq_{c}$ is the coupling frequency of *c*, and *s*_*c*_ is the coupling strength of *c*. $frq_{c}$ is ≥1 if there exists at least one *c* coupling from *u* to v, and 0 otherwise.

${\text{CCN}}_{\text{WD}}$ considers nine coupling types that might exist between two classes (suppose *u* and v are two classes in a system) [17,19]:

- Local VAriable (*LVA*): If *u* defines a method *m* which in turn defines a local variable of type v, then there is an LVA coupling from *u* to v.
- Global VAriable (*GVA*): If *u* defines a field *f* of type v, then there is a GVA coupling from *u* to v.
- INHeritance (*INH*): If *u* inherits v via keyword “extends", then there is an INH coupling from *u* to v.
- IMPlementation (*IMP*): If class *u* implements interface v via keyword “implements", then there is an IMP coupling from *u* to v.
- PARameter type (*PAR*): If *u* defines a method *m* that has a parameter of type v, then there is a PAR coupling from *u* to v.
- RETurn type (*RET*): If *u* defines a method *m* that has a return type v, then there is an RET coupling from *u* to v.
- INStantiates (*INS*): If *u* instantiates an object of v, then there is an INS coupling from *u* to v.
- ACCess (*ACC*): If one method *m* defined in *u* accesses a field *f* on an object of v, then there is an ACC coupling from *u* to v.
- MEthod Call (*MEC*): If one method *m*_1_ defined in *u* calls a method *m*_2_ on an object of v, then there is an MEC coupling from *u* to v.

Thus, *CS={LVA, GVA, IMP, PAR, RET, INS, ACC, MEC}*. According to Eq (2), to compute weights on links, we need to know *CS*, $frq_{c}$, and *s*_*c*_. Both *CS* and $frq_{c}$ can be resolved by static analysis of the source code. As for *s*_*c*_, we employ three ways to estimate its value [17]: Ordinal-scale-based Weighting Mechanism (OWM), Empirical Weighting Mechanism (EWM), and Distribution-based Weighting Mechanism (DWM). In OWM and EWM, the weight assigned to each coupling type is already shown in Table 3. In DWM, the weight for coupling type *c*, *s*_*c*_, is computed by

**Table 3:** Weights assigned by OWM and EWM [17,19].

OWM		EWM	
Weights	Coupling Types	Weights	Coupling Types
1	*MEC, PAR, LVA, RET*	4	*IMP*
5.5	*GVA*	3	*INH, PAR, RET, GVA*
7	*INH* (concrete parent)	2	*MEC, ACC, INS*
9	*INH* (abstract parent)	1	*LVA*
10	*IMP*	—	—
N/A	*ACC, INS*	—	—

$$s_{c}=\left\{ \begin{array}{cc} 10, & \mathrm{if}N_{\mathrm{intra}}^{c}\neq 0\wedge N_{\text{inter}}^{c}=0\\ 1, & \mathrm{if}N_{\text{intra}}^{c}=0\wedge N_{\text{inter}}^{c}=0\\ round(0.5+10\times \frac{N_{\text{intra}}^{c}}{N_{\text{intra}}^{c}+N_{\text{inter}}^{c}}), & \mathrm{otherwise} \end{array}\right.$$ (3)

where $N_{intra}^{𝑐}$ is the number of intra-package couplings of coupling *c*, and $N_{inter}^{𝑐}$ is the number of inter-package couplings. Intra-package couplings are the couplings occurring between two classes defined in the same package, while inter-package couplings are the couplings occurring between classes defined in two separate packages. round(y) returns an integer nearest to *y*.

Note that in OWM (cf. the left column of Table 3), the weight for both *ACC* and *INS* is “N/A", which means that OWM does not define weights for the two coupling types. Thus, when building ${\text{CCN}}_{\text{WD}}$s using OWM, we should discard the two coupling types. Interested readers can refer to Sect 4 of our online Appendix (https://github.com/SEGroupZJGSU/KCP/tree/main/Appendix) for a detailed description of the ${\text{CCN}}_{\text{WD}}$.

For our study, NMs is computed on ${\text{CCN}}_{\text{WD}}$s. But when computing NMs, we ignore weights on links, as NMs fits in with unweighted networks. For our study, all the metrics in NMs are computed using the Python package NetworkX (https://networkx.org/).

#### 2.2.3 Weighted network metric suite.

As pointed out in Sect 1, NMs does not consider the weight on links and thus it cannot reflect the actual complexity of classes. To tackle this problem, we build a weighted network metric suite — NMs_*w*_. NMs_*w*_ is actually a weighted variant of NMs. Thus, it also consists of 7 network metrics (see Table 2). Each metric in NMs_*w*_ corresponds to a metric in NMs. For example, *baryC*_*w*_ is the weighted variant of *baryC*. Note that all the weighted network metrics are collected from the existing research work in the field of complex networks [7,20,21]. In this work, we introduce their first application to KCP.

- **Weighted Barycenter Centrality** [22]
  In a weighted directed network, the *weighted barycenter centrality* of node *u*, ${baryC}_{w}(u)$, is defined as
  $$baryC_{w}(u)=\frac{1}{\sum_{u,v\in N\wedge u\neq v}wspl(u,v)},$$ (4)
  where *N* is the node set and wspl(u,v) is the weighted shortest path length from nodes *u* to v.
- **Weighted Betweenness Centrality** [23]
  In a weighted directed network, the *weighted betweenness centrality* of node *u*, ${betweenC}_{w}(u)$, is defined as
  $$betweenC_{w}(u)=\sum_{s,t,v\in N\wedge s\neq t\neq v}\frac{nwsp(s,t,u)}{nwsp(s,t)},$$ (5)
  where *N* is the node set, nwsp(s,t) is the number of weighted shortest paths between nodes *s* and *t*, and nwsp(s,t,u) is the number of weighted shortest paths between nodes *s* and *u* that pass through node *t*.
- **Weighted Closeness Centrality** [24]
  In a weighted directed network, the *weighted closeness centrality* of node *u*, ${closeC}_{w}(u)$, is defined as
  $$closeC_{w}(u)=\sum_{u,v\in N\wedge u\neq v}\frac{n-1}{wspl(u,v)},$$ (6)
  where *N* is the node set, *n* is the number of nodes in the network, and wspl(u,v) is the weighted shortest path length from nodes *u* to v.
- **Weighted Eigenvector Centrality** [20]
  In a weighted directed network, the *weighted eigenvector centrality* of node *u*, ${EigenC}_{w}(u)$, is the *u*-th element of the vector *x* defined by
  Ax=λx, (7)
  where *A* is the adjacency matrix of the network with eigenvalue *λ*.
- **Weighted Hub/Authority Scores** [7]
  In a weighted directed network, the *weighted hub scores* (${hub}_{w}(u)$) and *weighted authority scores* (${auth}_{w}(v)$) of nodes *u* and v are recursively computed by
  $$hub_{w}(u)=\sum_{u,v\in N\wedge u\neq v}\left(auth_{w}(v)\times w(u,v)\right),$$ (8)
  $$auth_{w}(v)=\sum_{u,v\in N\wedge u\neq v}\left(hub_{w}(u)\times w(u,v)\right),$$ (9)
  where *N* is the node set, and w(u,v) is the weight on the link from nodes *u* to v.
- **Weighted PageRank Values** [21]
  In a weighted directed network, the *weighted PageRank value* of node *u*, ${PR}_{w}(u)$, is computed by
  $$PR_{w}(u)=\frac{1-d}{m}+d\times \sum_{v\in inN(u)}\frac{PR_{w}(v)\times w(v,u)}{wOD(v)},$$ (10)
  where inN(u) is the in-neighbor of node *u*, w(v,u) is the weight on the link from nodes v to *u*, wOD(v) is the weighted out-degree of node v, and *m* is the number of nodes in the network. *d* is the damping factor (we use its default value 0.85).

For our study, NMs_*w*_ is computed on ${\text{CCN}}_{\text{WD}}$s, and all the metrics in NMs_*w*_ are computed using the Python package NetworkX (https://networkx.org/).

#### 2.2.4 Combined metric suite.

To examine whether a combination of different metric suites performs better than individual metric suites, we build four combined metric suites: DMs+NMs, DMs+NMs_*w*_, NMs+NMs_*w*_, and DMs+NMs+NMs_*w*_.

However, the *multicollinearity* problem may exist in the combined metric suites, which means that there are two or more independent variables with high linear correlations. The *multicollinearity* will affect the KCP models we built, as the effectiveness of some metrics on models may be masked by other collinear metrics. In this work, we compute the *Variance Inflation Factor* (*VIF*) of each metric in a combined metric suite to measure the degree of multicollinearity among metrics [25,26]. We remove all metrics with *VIF*>10, as previous studies found that metrics with *VIF*>10 are highly collinear with other metrics, and 10 has been a preferable cut-off value to deal with multicollinearity [26].

### 2.3 KCP contexts

For our study, we consider two KCP contexts: *within-project* context and *cross-project* context. The two contexts are differentiated from the source where the training data and testing data come from.

In the *within-project* context, the training data and testing data both come from the same version of a specific project. Generally, the data of a specific version of a project are divided into two parts: one for training a model, and the other for testing the trained model.

In the *cross-project* context, to predict key classes in the current version of a project (testing data), the training data are from other different projects. For example, to predict key classes in the project ArgoUML-0.9.5, a possible setting is to train the cross-project KCP model on the project Mars-3.06 (see Table 1).

### 2.4 Experiment setup

#### 2.4.1 Training and testing sets.

To ensure the statistical robustness of the obtained results, we used the *out-of-sample bootstrap sampling* technique [27], which randomly samples *M* observations with replacement from the data set of each project (suppose the data set has *M* observations) to create a training set. The observations that are not sampled as observations in the training set consist of a testing set. For each project, we resampled the data set 100 times to create 100 training sets and 100 testing sets. Thus, we can repeat our experiments 100 times for each project—once for each bootstrap sample.

The rationale to use *out-of-sample bootstrap sampling* technique rather than other sampling techniques such as cross-validation is twofold: i) Previous studies revealed that the bootstrap sampling procedure can ensure to obtain considerably more stable results for unseen observations [28]. ii) Our data set is high-skewed, as there are many more non-key classes than key ones. As shown in Table 1, in ten (≈55.56%) subject systems, the *imbalance rate* (i.e., *IR*) is less than 3%. Previous studies demonstrated that bootstrap sampling is suitable for high-skewed data sets [29].

#### 2.4.2 Data pre-processing.

In our data set, metrics are not always in the same order of magnitude. For example, the value of metrics in DMs is usually a non-negative integer, and the value of metrics in both NMs and NMs_*w*_ is usually in the range of [0,1). In the *within-project* KCP context, we normalized the data set using the *z-score* method [30], which can transform the value of each metric into a distribution with a *mean* of 0 and a standard deviation of 1. In the *cross-project* KCP context, we standardized the value of metrics using a *log transformation*, as the log transformation accounts for the *concept drift* commonly existing in the *cross-project* context [31]. Furthermore, in our data set, we labeled *key classes* as “1" and *non-key classes* as “0".

#### 2.4.3 Model construction.

In the *within-project* context, we used the Random Forest learner. We made such a choice mainly because [3] found that in the *within-project* context, Random Forest learner was an effective classification algorithm better than other eight classification algorithms, such as Decision Table, Decision Stumps, and J48 Decision Tree.

In the cross-project context, we used the Naive Bayes model [32], as [33] found that in the *cross-project* context, the Naive Bayes model performed best among 24 approaches identified in the literature.

We used the GridSearchCV (https://scikit-learn.org/stable/modules/generated/sklearn.model_selection.GridSearchCV.html) to tune the hyperparameters of the applied learners so as to ensure the built models can fit the data set well. In this work, all the prediction models are implemented using the Scikit-learn (Scikit-learn is publicly available at https://scikit-learn.org/) Python package. Specifically, we used the RandomForestClassifier (https://scikit-learn.org/stable/modules/generated/sklearn.ensemble.RandomForestClassifier.html) and GaussianNB (https://scikit-learn.org/stable/modules/generated/sklearn.naive_bayes.GaussianNB.html) functions to build the Random Forest learner and Naive Bayes model, respectively. Note that in this work, we did not use other classifiers, such as XGBoost, SVM, and deep learning methods, to build prediction models. The main reason is that our focus is on exploring the effectiveness of network metrics on the KCP, not on the performance of different classifiers.

#### 2.4.4 Performance metrics.

We used five performance metrics: *AUC* (Area Under the receiver-operator characteristic Curve) [34], *MCC* (Matthews Correlation Coefficient) [35], *Recall* [17], *Brier score* [36], and *Precision* [17].

*AUC* measures the area under the curve that plots the true positive rate against the false positive rate across all the thresholds.

*Brier score* measures the mean squared difference between the predicted probability and the actual probability assigned to a class, and is computed by

$$Brier\;score=\frac{1}{n}\sum_{i=1}^{n}{\left(p_{i}-y_{i}\right)}^{2},$$ (11)

where *p*_*i*_ and *y*_*i*_ are the predicted and actual probabilities for the *i*-th class, respectively; *y*_*i*_ = 1 if the *i*-th class is a key class, and *y*_*i*_ = 0 otherwise. *N* is the total number of classes.

The remaining three evaluation metrics can be defined as

$$MCC\;\;=\;\frac{TP\;\times \;TN\;-\;FP\;\times \;FN}{\sqrt{\left(TP\;\;+\;\;FP)(TP\;\;+\;\;FN)(TN\;\;+\;\;FP)(TN\;\;+\;\;FN\right)}},$$ (12)

$$Recall=\frac{TP}{TP+FN},$$ (13)

$$Precision=\frac{TP}{TP+FP},$$ (14)

where

- *TP* (True Positive) is the number of key classes that are also predicted as key classes.
- *FP* (False Positive) is the number of non-key classes that are predicted as key classes.
- *FN* (False Negative) is the number of key classes that are predicted as non-key classes.
- *TN* (True Negative) is the number of key classes that are also predicted as non-key classes.

For *Brier score*, a lower value indicates better performance. For the remaining metrics, a higher value indicates better performance.

#### 2.4.5 Model evaluation.

In a specific KCP context (i.e., *within-project* or *cross-project*), we performed the *Friedman test* with a *post-hoc Nemenyi test* [37] to determine the ranking of models (trained on different metric suites) in the whole set of projects. As we computed the performance of different models using five metrics, the Friedman test with a post-hoc Nemenyi test was performed metric-by-metric. However, the post-hoc Nemenyi test may return overlapping ranks, making the relative effectiveness of some metric suites indistinguishable. To avoid this situation, we, following the suggestion of [33], post-processed the results of the post-hoc Nemenyi test to generate a distinct *rankscore* for each model. The *rankscore* ranges between 0 and 1, and a larger *rankscore* indicates a better approach. We used such a ranking to determine the relative effectiveness of the considered metric suites on KCP. For example, if model *M*_1_ trained on NMs_*w*_ outperforms model *M*_2_ trained on NMs, then we can conclude that NMs_*w*_ is more effective than NMs on KCP.

In the *within-project* context, for a specific project, we obtained 100 performance values (as there are 100 bootstrap iterations) on a specific metric suite and performance metric. For our study, the Friedman test is performed on the median of the performance values obtained on the whole set of subject projects with respect to a performance metric. If the Friedman test returns a significant result, then we apply the post-hoc Nemenyi test and post-processing to get a non-overlapped ranking. In the *cross-project* context, we used a similar procedure as that in the *within-project* context. The main difference is that for a specific project, we will obtain 1,700 performance values (as there are 17 other subject projects and 100 bootstrap iterations) on a specific metric suite and performance metric.

We also performed the *Wilcoxon-signed rank test* [38] and *Cliff’s Delta effect size test* [39] to compare models trained on design metrics and other six metric suites (see Table 4 for example). Through the two tests, we can quantify the number of data sets where models trained on other six metric suites significantly (*p*-value < 0.05) perform better than that on design metrics with *effect size* larger than medium (i.e., the magnitude of Cliff’s Delta ≥0.33).

**Table 4:** The ranking (*rankscore*) results of our KCP models trained on different metric suites in the within-project context (organized by different weighting mechanisms).

Context	Weighting mechanisms	Evaluation metrics	Software metric suites						
			DMs	NMs	NMs *w*	DMs+NMs	DMs+NMs*w*	NMs+NMs*w*	DMs+NMs+NMs*w*
within-project	DWM	*AUC*	0.00	0.17 (5/18)^1^	0.50 (10/18)	0.34 (8/18)	0.67 (11/18)	0.84 (12/18)	1.00 (12/18)
		*MCC*	0.00	0.17 (5/18)	0.50 (8/18)	0.34 (5/18)	0.67 (9/18)	0.84 (9/18)	1.00 (10/18)
		*Recall*	0.00	0.17 (4/18)	0.50 (6/18)	0.34 (3/18)	0.67 (6/18)	0.84 (9/18)	1.00 (9/18)
		*Brier score*	0.00	0.17 (5/18)	0.50 (9/18)	0.34 (5/18)	0.67 (10/18)	0.84 (10/18)	1.00 (10/18)
		*Precision*	0.17	0.00 (3/18)	0.50 (7/18)	0.34 (4/18)	0.84 (6/18)	0.67 (7/18)	1.00 (7/18)
	OWM	*AUC*	0.00	0.17 (5/18)	0.50 (10/18)	0.34 (8/18)	0.67 (13/18)	0.84 (13/18)	1.00 (14/18)
		*MCC*	0.00	0.17 (5/18)	0.50 (9/18)	0.34 (3/18)	0.67 (8/18)	0.84 (9/18)	1.00 (9/18)
		*Recall*	0.00	0.17 (4/18)	0.50 (8/18)	0.34 (2/18)	0.67 (7/18)	1.00 (8/18)	0.84 (8/18)
		*Brier score*	0.00	0.17 (3/18)	0.50 (9/18)	0.34 (5/18)	0.67 (9/18)	0.84 (10/18)	1.00 (10/18)
		*Precision*	0.17	0.00 (3/18)	0.50 (6/18)	0.34 (1/18)	0.84 (5/18)	0.67 (6/18)	1.00 (6/18)
	EWM	*AUC*	0.00	0.17 (5/18)	0.50 (9/18)	0.34 (7/18)	0.67 (11/18)	0.84 (13/18)	1.00 (12/18)
		*MCC*	0.00	0.17 (4/18)	0.50 (9/18)	0.34 (3/18)	0.67 (10/18)	0.84 (10/18)	1.00 (10/18)
		*Recall*	0.00	0.17 (4/18)	0.50 (8/18)	0.34 (2/18)	0.67 (7/18)	0.84 (10/18)	1.00 (9/18)
		*Brier score*	0.00	0.17 (4/18)	0.50 (9/18)	0.34 (5/18)	0.67 (9/18)	0.84 (10/18)	1.00 (10/18)
		*Precision*	0.17	0.00 (3/18)	0.50 (7/18)	0.34 (4/18)	0.84 (7/18)	0.67 (7/18)	1.00 (6/18)

^1^ The values in each cells are shown in the form of “r(x/y)", where *r* denotes the *rankscore* of the model trained on different metric suites, *x* is the number of data sets (the data for each system constitute a separate data set) where the model trained on the metric suite signified by the column name significantly outperforms (i.e., *p*-value <0.05) the model trained on DMs with *effect size* larger than medium (i.e., the magnitude of Cliff’s Delta ≥0.33), and *y* is the total number of data sets.

## 3 Results and analysis

We adhere to the *experiment setup* described in Sect 2.4 to perform the experiments. In this section, we present and analyze the obtained results that are related to the research questions raised in Sect 1.

### 3.1 (RQ1) Do network metrics outperform design metrics in the within-project KCP context?

#### 3.1.1 Results.

The ranking (or *rankscore*) results of our KCP models trained on different metric suites in the within-project context are presented in Table 4. The *p*-value of all Friedman tests is ≪0.05, which indicates that the models trained on different metric suites are different from each other. In this section, we present some observations derived from these results.

**Observation** 1: *In the within-project context, models trained on NMs and NMs*_*w*_ *are all superior to models trained on DMs, which indicates that network metrics (i.e., NM and NMs*_*w*_*) are more effective than design metrics on KCP.* Specifically, from Table 4, we observe that when the weighting mechanism is DWM, i) the *rankscore* of models trained on NMs is larger than that on DMs for four out of the five performance metrics (the only exception is *precision*); ii) the *rankscore* of models trained on NMs_*w*_ is larger than that on DMs as per all the five performance metrics; iii) the number of data sets where models trained on NMs outperform models trained on DMs with an effect size larger than medium is small (at most 5 out of the 18 data sets); and iv) on at most 10 out of the 18 data sets, the model trained on NMs_*w*_ is significantly better than that on DMs with an effect size larger than medium. In other two weighting mechanisms, the same findings have also been noted.

**Observation** 2: *In the within-project context, models trained on DMs+NMs, DMs+NMs*_*w*_*, NMs+NMs*_*w*_*, and DMs+NMs+NMs*_*w*_ *all surpass models trained on their separate components (i.e., DMs, NMs, and NMs*_*w*_*), which indicates that combined metric suites do better than their separate components on KCP.* As shown in Table 4, in all the three weighting mechanisms, the *rankscore* of models trained on DMs+NMs is 0.34, larger than that on DMs or NMs alone for all the five performance metrics, which are 0.00 and 0.17, respectively. Similar findings can also be noted in metric suites DMs+NMs_*w*_, NMs+NMs_*w*_, and DMs+NMs+NMs_*w*_.

**Observation** 3: *Models trained on NMs*_*w*_ *alone or along with DMs and/or NMs outperform models trained on NMs alone or along with DMs, which indicates that weighted network metrics can improve the performance of models based on unweighted network metrics.* We observe from Table 4 that, in all the three weighting mechanisms, i) the *rankscore* of models trained on NMs_*w*_ is 0.5, larger than that on NMs for all the five performance metrics, which is 0.17 (0.00 for *Precision*); ii) the *rankscore* of models trained on DMs+NMs_*w*_ is larger than that on DMs+NMs for all the five performance metrics, which is 0.34; iii) the *rankscore* of models trained on NMs+NMs_*w*_ is larger than that on NMs for all the five performance metrics, which is 0.17 (0.00 for *Precision*); and iv) the *rankscore* of models trained on DMs+NMs+NMs_*w*_ is larger than that on DMs+NMs for all the five performance metrics, which is 0.34.

**Observation** 4: *In the within-project context, different weighting mechanisms seem to have very small effect on the relative effectiveness of modes trained on different metric suites.* From Table 4, we observe that in the three weighting mechanisms, models trained on the seven metric suites can be roughly sorted into the following order w.r.t. their *rankscore* values for all the five performance metrics: DMs+NMs+NMs_*w*_, DMs+NMs_*w*_, NMs+NMs_*w*_, NMs_*w*_, DMs+NMs, NMs, and DMs, which means the model trained on DMs+NMs+NMs_*w*_ performs best, and the model trained on DMs performs worst. Note that the order is determined by counting the number of times that one metric suite performs better than the other. For example, there are 15=3×5 (i.e., 3 weighting mechanisms and 5 performance metrics) cases in total; DMs+NMs+NMs_*w*_ outperforms DMs+NMs_*w*_ on 14/15 of the cases.

**Observation** 5: *In the within-project context, among all the combinations of metric suites and weighting mechanisms for KCP, the best combination does not keep the same across different performance metrics.* Table 5 shows the ranking results of models trained on different combinations of metrics suites and weighting mechanisms; the ranking results are organized according to different performance metrics. The *p*-value of all Friedman tests is ≪0.05, which indicates that the models trained on different metric suites are different from each other. From Table 5, we observe that i) DMs+NMs+NMs_*w*_ along with EWM performs best for *AUC*, *MCC*, and *Recall*, ii) DMs+NMs+NMs_*w*_ along with DWM performs best for *Precision*, and iii) DMs+NMs+NMs_*w*_ along with OWM performs best for *Brier score*. Overall, DMs+NMs+NMs_*w*_ along with EWM performs best across the five performance metrics (cf. the gray-colored cells in Table 5).

**Table 5:** The ranking (*rankscore*) results of our KCP models trained on different metric suites in the within-project context (in the whole set of three weighting mechanisms).

Context	Weighting mechanisms	Metric suites	Rankings				
			*AUC*	*MCC*	*Recall*	*Brier score*	*Precision*
within-project	DWM	DMs	0.00	0.00	0.05	0.00	0.20
		NMs	0.20	0.15	0.15	0.20	0.05
		NMs_*w*_	0.55	0.45	0.45	0.50	0.50
		DMs+NMs	0.30	0.35	0.30	0.30	0.40
		DMs+NMs_*w*_	0.60	0.60	0.55	0.60	0.80
		NMs+NMs_*w*_	0.80	0.65	0.75	0.75	0.65
	OWM	DMs+NMs+NMs_*w*_	0.90	0.90	0.80	0.90	1.00
		DMs	0.05	0.05	0.10	0.10	0.25
		NMs	0.25	0.25	0.25	0.25	0.00
		NMs_*w*_	0.50	0.55	0.60	0.55	0.45
		DMs+NMs	0.40	0.30	0.35	0.40	0.30
		DMs+NMs_*w*_	0.70	0.80	0.70	0.65	0.75
		NMs+NMs_*w*_	0.75	0.85	0.95	0.85	0.60
		DMs+NMs+NMs_*w*_	0.95	0.95	0.90	1.00	0.85
	EWM	DMs	0.10	0.10	0.00	0.05	0.15
		NMs	0.15	0.20	0.20	0.15	0.10
		NMs_*w*_	0.45	0.50	0.50	0.45	0.55
		DMs+NMs	0.35	0.40	0.40	0.35	0.35
		DMs+NMs_*w*_	0.65	0.70	0.65	0.70	0.90
		NMs+NMs_*w*_	0.85	0.75	0.85	0.80	0.70
		DMs+NMs+NMs_*w*_	1.00	1.00	1.00	0.95	0.95

#### 3.1.2 Analysis.

From the results shown in Sect 3.1.1, we observe that in the *within-project* KCP context, i) network metrics outperform design metrics, and ii) weighted network metrics are better than unweighted network metrics. We hypothesize that such a result might be because i) network metrics are more relevant to key classes than design metrics, and ii) weighted network metrics are more relevant to key classes than unweighted network metrics. We arrive at such a hypothesis as Ref [5] found that unweighted network metrics can discriminate key classes better than design metrics and thus can be used to improve the performance of design metrics based KCP models.

To find the most discriminative metrics in the whole set of metric suites for KCP, we computed the *information gain* (*InfoGain*) [40,41] for each metric and used the *InfoGain* to measure the relevance of each metric for key classes. Generally, a metric with a larger *InfoGain* value indicates it is a more discriminative metric. For our study, we first computed the *InfoGain* for each metric system-by-system. Then, we ranked all the metrics in descending order according to their *InfoGain* values, and the top-ranked metrics are treated as the most discriminative ones.

For illustration purpose, Table 6 shows the ranking results (in descending order) of all considered metrics according to their *InfoGain* values obtained on the first nine projects in Table 1 (the weighting mechanism is DWM). The ranking results obtained on each project can be found in our online replication package (https://github.com/SEGroupZJGSU/KCP/tree/main/IG). From Table 6, we observe that i) metrics in NMs_*w*_ can discriminate key classes better than NMs (with only two exceptions when metrics are *hub* and *PR*), and ii) metrics in NMs can discriminate key classes better than DMs. Such observations can partially explain the improvement made by network metrics.

**Table 6:** The ranking results (*rankscore* in descending order) of different metrics according to their information gain obtained on the first 9 projects in Table 1 (The weighting mechanism is DWM).

Metric suites	ArgoUML	Mars	javaclient	JGAP	Neuroph	JPMC	wro4j	xuml-compiler	Maze
NMs_*w*_	*hub*_*w*_	*PR*_*w*_	*PR*_*w*_	*PR*_*w*_	*PR*_*w*_	*PR*_*w*_	*PR*_*w*_	*PR*_*w*_	*PR*_*w*_
	*betweenC*_*w*_	*EigenC*_*w*_	*EigenC*_*w*_	*hub*_*w*_	*EigenC*_*w*_	*EigenC*_*w*_	*EigenC*_*w*_	*auth*_*w*_	*EigenC*_*w*_
	*EigenC*_*w*_	*auth*_*w*_	*auth*_*w*_	*EigenC*_*w*_	*auth*_*w*_	*hub*_*w*_	*auth*_*w*_	*EigenC*_*w*_	*auth*_*w*_
	*auth*_*w*_	*hub*_*w*_	*hub*_*w*_	*auth*_*w*_	*closeC*_*w*_	*auth*_*w*_	*closeC*_*w*_	*hub*_*w*_	*hub*_*w*_
	*PR*_*w*_	*closeC*_*w*_	*closeC*_*w*_	*baryC*_*w*_	*hub*_*w*_	*closeC*_*w*_	*hub*_*w*_	*closeC*_*w*_	*closeC*_*w*_
	*closeC*_*w*_	*baryC*_*w*_	*baryC*_*w*_	*closeC*_*w*_	*baryC*_*w*_	*baryC*_*w*_	*baryC*_*w*_	*baryC*_*w*_	*baryC*_*w*_
	*baryC*_*w*_	mygray*hub*	mygray*hub*	mygray*hub*	mygray*PR*	mygray*PR*	mygray*PR*	mygray*hub*	mygray*hub*
NMs	*hub*	*PR*	*PR*	*PR*	*hub*	*hub*	mygray*betweenC*_*w*_	*PR*	*PR*
	*PR*	mygray*betweenC*_*w*_	*EigenC*	*EigenC*	*EigenC*	*EigenC*	*auth*	*EigenC*	*EigenC*
	*auth*	*auth*	*auth*	mygray*betweenC*_*w*_	mygray*betweenC*_*w*_	mygray*betweenC*_*w*_	*hub*	mygray*betweenC*_*w*_	mygray*betweenC*_*w*_
	*EigenC*	*EigenC*	mygray*betweenC*_*w*_	*auth*	*auth*	*auth*	*EigenC*	*auth*	*auth*
	*closeC*	*closeC*	*closeC*	*closeC*	*closeC*	*closeC*	*closeC*	*closeC*	*closeC*
	*betweenC*	*betweenC*	*baryC*	*baryC*	*betweenC*	*betweenC*	*betweenC*	*baryC*	*baryC*
	*baryC*	*baryC*	*betweenC*	*betweenC*	*baryC*	*baryC*	*baryC*	*betweenC*	*betweenC*
DMs	*IC_Par*	*Dep_In*	*Dep_In*	*Dep_In*	*Dep_In*	*Dep_In*	*Dep_In*	*Dep_In*	*Dep_In*
	*NumPubOps*	*EC_Par*	*EC_Par*	*NumOps*	*EC_Par*	*NumPubOps*	*EC_Par*	*EC_Par*	*EC_Par*
	*NumOps*	*NumPubOps*	*NumPubOps*	*EC_Par*	*NumPubOps*	*NumOps*	*NumPubOps*	*NumPubOps*	*NumPubOps*
	*Dep_In*	*NumOps*	*NumOps*	*NumPubOps*	*NumOps*	*EC_Par*	*NumOps*	*NumOps*	*NumOps*
	*EC_Par*	*Getters*	*IC_Par*	*Getters*	*EC_Attr*	*IC_Par*	*EC_Attr*	*Getters*	*EC_Attr*
	*NumAttr*	*IC_Par*	*NumAttr*	*IC_Par*	*Getters*	*Getters*	*Getters*	*IC_Par*	*Getters*
	*EC_Attr*	*EC_Attr*	*Getters*	*NumAttr*	*IC_Par*	*NumAttr*	*IC_Par*	*EC_Attr*	*IC_Par*
	*Getters*	*NumAttr*	*EC_Attr*	*Setters*	*NumAttr*	*EC_Attr*	*Setters*	*Setters*	*NumAttr*
	*Dep_Out*	*Setters*	*IC_Attr*	*EC_Attr*	*Setters*	*Setters*	*NumAttr*	*NumAttr*	*Setters*
	*IC_Attr*	*Dep_Out*	*Dep_Out*	*Dep_Out*	*Dep_Out*	*Dep_Out*	*Dep_Out*	*Dep_Out*	*Dep_Out*
	*Setters*	*IC_Attr*	*Setters*	*IC_Attr*	*IC_Attr*	*IC_Attr*	*IC_Attr*	*IC_Attr*	*IC_Attr*

***The answer to RQ1:** In the within-project context, network metrics (i.e., NMs and NMs*_*w*_*) alone or along with design metrics (i.e., DMs) are more effective than DMs alone on KCP; NMs*_*w*_ *is superior to both NMs and NMs+DMs. The above observations hold for all the three considered weighting mechanisms. Furthermore, EWM and DMs+NMs+NMs*_*w*_ *can train a model that achieves the best performance across five performance metrics.*

### 3.2 (RQ2) Do network metrics outperform design metrics in the cross-project KCP context?

#### 3.2.1 Results.

Table 7 shows the ranking (or *rankscore*) results of our KCP models trained on different metric suites in the cross-project context. The *p*-value of all Friedman tests is ≪0.05, which indicates that the models trained on different metric suites are different from each other. In this section, we describe the observations derived from the results.

**Table 7:** The ranking (*rankscore*) results of our KCP models trained on different metric suites in the cross-project context (organized by different weighting mechanisms).

Context	Weighting mechanisms	Evaluation metrics	Software metric suites						
			DMs	NMs	NMs *w*	DMs+NMs	DMs+NMs*w*	NMs+NMs*w*	DMs+NMs+NMs*w*
cross-project	DWM	*AUC*	1.00	0.34 (0/18)^1^	0.00 (0/18)	0.84 (0/18)	0.67 (0/18)	0.17 (0/18)	0.50 (0/18)
		*MCC*	0.84	0.34 (0/18)	0.00 (0/18)	1.00 (0/18)	0.50 (0/18)	0.17 (0/18)	0.67 (0/18)
		*Recall*	1.00	0.67 (3/18)	0.00 (0/18)	0.84 (2/18)	0.34 (0/18)	0.17 (0/18)	0.50 (0/18)
		*Brier score*	0.17	0.34 (3/18)	0.84 (3/18)	0.67 (2/18)	1.00 (3/18)	0.00 (1/18)	0.50 (5/18)
		*Precision*	0.84	0.67 (0/18)	0.17 (0/18)	1.00 (0/18)	0.34 (0/18)	0.00 (0/18)	0.50 (0/18)
	OWM	*AUC*	1.00	0.00 (0/18)	0.17 (0/18)	0.84 (0/18)	0.67 (0/18)	0.34 (0/18)	0.50 (0/18)
		*MCC*	1.00	0.00 (0/18)	0.34 (1/18)	0.84 (0/18)	0.67 (1/18)	0.17 (1/18)	0.50 (1/18)
		*Recall*	1.00	0.17 (4/18)	0.00 (1/18)	0.84 (2/18)	0.67 (1/18)	0.34 (1/18)	0.50 (1/18)
		*Brier score*	0.17	0.34 (2/18)	1.00 (6/18)	0.67 (2/18)	0.84 (4/18)	0.00 (4/18)	0.50 (5/18)
		*Precision*	0.84	0.34 (0/18)	0.17 (1/18)	1.00 (1/18)	0.67 (2/18)	0.00 (1/18)	0.50 (1/18)
	EWM	*AUC*	1.00	0.17	0.00 (0/18)	0.84 (0/18)	0.67 (0/18)	0.34 (0/18)	0.50 (0/18)
		*MCC*	1.00	0.00 (0/18)	0.17 (1/18)	0.84 (0/18)	0.67 (1/18)	0.34 (1/18)	0.50 (1/18)
		*Recall*	1.00	0.34 (4/18)	0.00 (1/18)	0.84 (2/18)	0.50 (1/18)	0.17 (1/18)	0.67 (1/18)
		*Brier score*	0.00	0.17 (2/18)	1.00 (7/18)	0.34 (2/18)	0.84 (5/18)	0.50 (5/18)	0.67 (5/18)
		*Precision*	0.84	0.50 (0/18)	0.17 (1/18)	1.00 (1/18)	0.67 (1/18)	0.00 (1/18)	0.34 (1/18)

^1^ The values in each cells are shown in the form of “r(x/y)", where *r* denotes the *rankscore* of the model trained on different metric suites, *x* is the number of data sets where the model trained on the metric suite signified by the column name significantly outperforms (i.e., *p*-value <0.05) the model trained on DMs with *effect size* larger than medium (i.e., the magnitude of Cliff’s Delta ≥0.33), and *y* is the total number of data sets.

**Observation** 1: *In the cross-project context, models trained on design metrics (i.e., DMs) are roughly better than models trained on NMs or NMs*_*w*_ *alone on KCP.* As shown in Table 7, we observe that when the ${\text{CCN}}_{\text{WD}}$ is built using the DWM weighting mechanism, the *rankscore* of models trained on DMs is larger than that trained on NMs (or NMs_*w*_) for four out of the five performance metrics. The only exception is *Brier score*, where NMs and NMs_*w*_ are both better than DMs on KCP. However, the number of data sets on which models trained on NMs (or NMs_*w*_) outperform models trained on DMs with an effect size larger than medium is small (at most 3 out of the 18 data sets). In other two weighting mechanisms, we can make the same observations.

**Observation** 2: *In the cross-project context, models trained on network metrics (i.e., NMs or NMs*_*w*_*) along with DMs do better than models trained on network metrics alone (i.e., NMs or NMs*_*w*_*) on KCP.* From Table 7, it can be seen that in all the three weighting mechanisms, the *rankscore* of models trained on DMs+NMs (or DMs+NMs_*w*_) is larger than that trained on NMs (or NMs_*w*_) alone for all the five performance metrics.

**Observation** 3: *In the cross-project context, the performance of models trained on NMs and NMs*_*w*_ *do not have a clear difference, but models trained on DMs+NMs outperform models trained on DMs+NMs*_*w*_. It can be seen from Table 7 that i) when the weighting mechanism is DWM, models trained on NMs perform better than models trained on NMs_*w*_ for four performance metrics (the only exception is *Brier score*), ii) when the weighting mechanism is OWM, models trained on NMs_*w*_ outperform models trained on NMs for three performance metrics (the two exceptions are *Recall* and *Precision*), iii) when the weighting mechanism is EWM, models trained on NMs are better than models trained on NMs_*w*_ for three performance metrics (the two exceptions are *MCC* and *Brier score*), and iv) in the three weighting mechanisms, models trained on DMs+NMs do better than models trained on DMs+NMs_*w*_ only with three exceptions when performance metric is *Brier score*.

**Observation** 4: *In the cross-project context, different weighting mechanisms have a slight impact on the relative effectiveness of modes trained on different metric suites.* As shown in Table 7, we find that the ranking results of models trained on the same set of metric suites do not keep the same across different weighting mechanisms. Specifically, i) when the weighting mechanism is DWM, models trained on the seven metric suites can be roughly sorted into the following order: DMs+NMs, DMs, DMs+NMs+NMs_*w*_, DMs+NMs_*w*_, NMs, NMs+NMs_*w*_, and NMs_*w*_, which means the model trained on DMs+NMs performs best, and the model trained on NMs_*w*_ performs worst; ii) when the weighting mechanism is OWM, models trained on the seven metric suites can be roughly sorted into the following order: DMs, DMs+NMs, DMs+NMs_*w*_, DMs+NMs+NMs_*w*_, NMs_*w*_, NMs+NMs_*w*_, and NMs; and iii) when the weighting mechanism is EWM, models trained on the seven metric suites can be roughly sorted into the following order: DMs, DMs+NMs, DMs+NMs_*w*_, DMs+NMs+NMs_*w*_, NMs+NMs_*w*_, NMs, and NMs_*w*_. Note that the above rankings are determined by counting the number of times that one metric suite performs better than the other according to the five performance metrics. For example, when the weighting mechanism is DWM, there are 5 (i.e., 5 performance metrics) cases in total; DMs+NMs outperforms DMs on 3/5 of the cases when performance metrics are *MCC*, *Brier score*, and *Precision*.

**Observation** 5: *In the cross-project context, among all the combinations of metric suites and weighting mechanisms for KCP, the best combination does not remain consistent across different performance metrics.* Table 8 shows the ranking results of models trained on different combinations of metrics suites and weighting mechanisms, where the ranking results are organized according to different performance metrics. The *p*-value of all Friedman tests is ≪0.05, which indicates that the models trained on different metric suites are different from each other. From Table 8, we observe that DMs along with OWM performs best for *AUC*, NMs_*w*_ along with EWM performs best for *Brier score*, and DMs along with EWM performs best for *MCC*, *Recall*, and *Precision* (cf. the gray-colored cells in Table 8). Overall, it seems that DMs along with EWM performs best across the five performance metrics (cf. the gray-colored cells in Table 8).

**Table 8:** The ranking (*rankscore*) results of our KCP models trained on different metric suites in the cross-project context (in the whole set of three weighting mechanisms).

Context	Weighting mechanisms	Metric suites	Rankings				
			*AUC*	*MCC*	*Recall*	*Brier score*	*Precision*
cross-project	DWM	DMs	0.95	0.90	0.95	0.35	0.95
		NMs	0.25	0.20	0.35	0.20	0.55
		NMs_*w*_	0.05	0.00	0.00	0.70	0.05
		DMs+NMs	0.75	0.75	0.75	0.60	0.80
		DMs+NMs_*w*_	0.50	0.40	0.15	0.75	0.10
		NMs+NMs_*w*_	0.00	0.05	0.10	0.00	0.00
		DMs+NMs+NMs_*w*_	0.45	0.50	0.20	0.45	0.20
	OWM	DMs	1.00	0.95	0.90	0.25	0.75
		NMs	0.20	0.15	0.45	0.10	0.60
		NMs_*w*_	0.35	0.45	0.25	0.95	0.40
		DMs+NMs	0.85	0.85	0.85	0.50	0.85
		DMs+NMs_*w*_	0.70	0.70	0.70	0.80	0.70
		NMs+NMs_*w*_	0.40	0.35	0.60	0.05	0.25
	EWM	DMs+NMs+NMs_*w*_	0.65	0.65	0.65	0.40	0.65
		DMs	0.90	1.00	1.00	0.30	1.00
		NMs	0.15	0.10	0.40	0.15	0.45
		NMs_*w*_	0.10	0.25	0.05	1.00	0.30
		DMs+NMs	0.80	0.80	0.80	0.55	0.90
		DMs+NMs_*w*_	0.60	0.60	0.50	0.90	0.50
		NMs+NMs_*w*_	0.30	0.30	0.30	0.65	0.15
		DMs+NMs+NMs_*w*_	0.55	0.55	0.55	0.85	0.35

#### 3.2.2 Analysis.

From the results shown in Sect 3.2.1, we observe that in the cross-project KCP context, i) design metrics are superior to network metrics (i.e., NMs or NMs_*w*_ alone), and ii) design metrics along with network metrics (i.e., NMs or NMs_*w*_) outperform network metrics alone (i.e., NMs or NMs_*w*_ alone). Such observations contradict the results that we found in Sect 3.1.1. We hypothesize that such a result might come from i) design metrics are less project-specific and thus are more suitable to build cross-project KCP models, and ii) network metrics are more project-specific and thus are more suitable to build within-project KCP models. We arrive at such a hypothesis as for less project-specific metrics, their values across different projects might be more similar to each other. Thus, KCP models trained for one project might have higher probability to be applicable to other projects.

To find the least project-specific metric suites for KCP in the cross-project context, we performed the *one-way ANOVA test* metric-by-metric and computed the *F-score* for each metric through dividing the *variance between projects* by the *variance within the projects*. In the *one-way ANOVA test*, a large *F-score* usually indicates that there are large differences between the means of the metric computed on different projects, and thus the metric tends to be more project-specific. On the contrary, a small *F-score* usually suggests that there are small differences between the means of the metric computed on different projects, and thus the metric tends to be less project-specific. For our study, we first aggregated the value data of a specific metric computed on all the subject projects to build a separate data set. Then, we applied the *one-way ANOVA test* on such a data set to obtain the *F-score* for this metric (we compute the *F-score* for all the metrics by a similar way). After that, we computed the median *F-score* for each metric suite. Finally, we ranked all the metric suites in ascending order according to their median *F-score* values, and the top-ranked metric suites tend to be less project-specific.

Table 9 shows the median *F-score* of all considered metric suites obtained on the whole set of subject systems (the complete results are available at https://github.com/SEGroupZJGSU/KCP/tree/main/F-score). From Table 9, we observe that i) the median *F-score* value of DMs is much smaller than that of both NMs and NMs_*w*_, which indicates that compared with NMs and NMs_*w*_, DMs is less project-specific; and ii) the median *F-score* values of DMs+NMs and DMs+NMs_*w*_ are much smaller than that of NMs and NMs_*w*_, respectively. Such observations can partially explain the observations made in Sect 3.2.1.

**Table 9:** The median *F-score* for each metric suite (organized by different weighting mechanisms).

Context	Weighting mechanisms	*F-score* of different metric suites						
		DMs	NMs	NMs_*w*_	DMs+NMs	DMs+NMs_*w*_	NMs+NMs_*w*_	DMs+NMs+NMs_*w*_
cross-project	DWM	11.22	62.84	44.62	15.04	13.57	51.18	15.98
	OWM	11.22	62.84	45.02	15.04	14.75	52.47	15.98
	EWM	11.22	62.84	45.21	15.04	13.57	54.02	15.98

***The answer to RQ2:** In the cross-project KCP context, i) design metrics (i.e., DMs) outperform network metrics (i.e., NMs or NMs*_*w*_ *alone); ii) network metrics (i.e., NMs or NMs*_*w*_*) along with design metrics do better than network metrics alone (i.e., NMs or NMs*_*w*_*); and iii) weighting mechanisms have slight impact on the relative effectiveness of different metric suites on KCP. Furthermore, the best model does not remain consistent across different performance metrics.*

## 4 Threats to validity

In this section, we discuss the main threats that might affect the construct, internal, and external validity of our work.

**Threats to Construct Validity.** The main threat to the construct validity of our work is the accuracy of the independent and dependent variables. The independent variables in this work are both design metrics (i.e., DMs) and network metrics (i.e., NMs and NMs_*w*_). To collect the design metrics, we used two mature commercial tools — MagicDraw 16.5 and SDMetrics V2.5. To collect the network metrics, we used SNAP to build software networks and implemented a Python script using NetworkX [42] to compute metric values. SNAP has been thoroughly tested in the past nine years and also has been used many times to build software networks [2,8,14,17,30,43]. NetworkX is a Python package widely used in previous software engineering literature [44–47]. To ensure the quality of the obtained metric values, the Python script has been thoroughly tested by the first and second authors. The dependent variable in this work is a binary variable (i.e., the label) signifying a class is a key class or not. For all the 18 subject systems, the label information is directly borrowed from the literature on key class identification [7,8,11–15,17], where the key classes are identified from free texts or pruned diagrams in the design documentation through phrases like *architectural overview* and *core of the system*. To ensure the quality of the label information, we doubly checked the key classes for each subject system following the process provided in Ref [7]. Thus, we believe that the construct validity of both the dependent and independent variables in this work can be considered satisfactory.

**Threats to Internal Validity.** The first threat to the internal validity of our study is related to the metrics that we used to train KCP models. As shown in Table 2, we used 11 design metrics (i.e., DMs), 7 unweighted network metrics (i.e., NMs), and 7 weighted network metrics (i.e., NMs_*w*_) to train models that predict key classes. DMs and NMs are both from previous studies [5], and NMs_*w*_ is a weighted version of NMs. These metrics may affect the performance of KCP models and the conclusions obtained in this work. We selected all the metric suites (i.e., DMs and NMs) used in the KCP field. However, other metric suites that we have ignored may also have the ability to improve the performance of KCP models, such as the CK metric suite [48], Bansiy and Davis’s metric suite [49], and McCabe’s metric suite [50]. But it is not the focus of this work. In this work, our focus is to investigate the effectiveness of network metrics on KCP — that is, the relative effectiveness of network metrics over design metrics. We plan to explore the effectiveness of other OO metrics on KCP in the future.

The second threat is related to the machine-learning techniques we used to train KCP models. As described in Sect 2.4.3, we used the Random Forest learner in the within-project context, and used the NavieBayes model in the cross-project context. The used techniques may affect the performance of KCP models and our conclusions. We chose the two techniques following the suggestions given by two previous studies [3,33]. In fact, it is not the focus of this work to investigate the performance of different classification techniques on KCP. We plan to explore the impact of different classification techniques on KCP and consider applying more machine learning techniques to the KCP in the future [51,52].

**Threats to External Validity.** The main threat to the external validity of our study is that our conclusions suffer from the threat to be generalized to other systems. As shown in Table 1, we used a set of 18 projects as research subjects, varying in size and domains. It is the largest data set used in the KCP literature so far, which is large enough to draw statistically meaningful conclusions. However, these systems are all open-source Java projects. Thus, our conclusions suffer from the threat to be generalized either to systems not developed in Java or to systems that are closed-source. In fact, this is a problem that most studies in empirical software engineering may face. To alleviate this threat, in the future, we will replicate our study on both systems developed in other programming languages and systems that are closed-source.

## 5 Implications for software engineering

In this section, we will discuss the theoretical and practical value of the research methods proposed in this paper.

- In traditional software comprehension processes, developers often rely on reverse engineered class diagrams to aid understanding. However, due to the large scale of systems and complex dependency relationships, the generated class diagrams often contain a significant amount of redundant information, such as non-core classes, low-coupling modules, or deprecated code structures. This not only increases the cognitive burden on developers but may also obscure critical design logic. Our approach helps retain the classes and relationships that are crucial for understanding the system architecture, enabling developers to quickly focus on core designs and shorten the time required for reverse analysis. Developers can rapidly locate modules that need modification or extension based on these key classes. Additionally, new team members can grasp the core logic of the system more quickly through an intuitive class diagram, reducing the cognitive overhead of team collaboration.
- High-quality code documentation is a critical factor in enhancing software maintainability, particularly during the development and maintenance of large-scale complex systems, where its importance becomes even more pronounced. However, in practical development scenarios, due to constraints in time, resources, or manpower, development teams often struggle to document all code modules with equal thoroughness. Therefore, adopting a priority-driven documentation strategy becomes particularly essential—that is, focusing on thorough documentation for modules that influence the architectural core, bear critical functionalities, or are frequently invoked (i.e., key classes), while simplifying descriptions for less critical modules. Our approach provides a scientific foundation for such prioritization decisions, effectively improving development efficiency while reducing maintenance costs. Furthermore, our enhanced software network representation enables more accurate key class prediction. In practical applications, appropriate network metrics can be selectively employed to predict key classes based on different software system types and specific operational contexts.
- Our approach may also be helpful in the personalized comprehension of a software system. When developers, especially new members, face large-scale systems, they often struggle to quickly locate the core logic due to the overwhelming size of the code. By predicting key classes—such as architectural core classes or frequently invoked classes—it becomes possible to directly highlight the most critical modules for global understanding, enabling developers to ignore redundant details. Different roles within a development team, such as maintainers, new developers, and architects, have varying focuses when it comes to the system. By integrating our approach with developer context (e.g., current tasks or historical operations), we can dynamically adjust key class recommendations to better suit their specific needs.

## 6 Related work

Our goal is not to propose yet another key-class predictor, but to conduct the first systematic investigation of how different network metrics influence KCP performance. Because, to the best of our knowledge, no prior work has examined this question, this section concentrates on surveying existing work on detecting key classes in software systems, which can be roughly grouped into two categories: *supervised approaches* (aka KCP approaches) and *unsupervised approaches*. *Supervised approaches* usually applied machine-learning techniques to train classification models to predict key classes. *Unsupervised approaches* do not rely on any machine-learning techniques. They usually proposed some metrics to quantify class importance and treated the top-ranked classes as key class candidates.

### 6.1 Supervised approaches (KCP approaches)

Ref [3] used a set of design metrics to characterize the properties of classes, and then trained several machine-learning classifiers on these metrics to predict key classes in a system. Ref [5] improved Osman et al.’s work by further considering the unweighted network metrics. They found that the combination of both design metrics and unweighted network metrics can improve the performance of KCP models. Ref [10] proposed a machine-learning based approach, MCCondenser, to identify key classes. Their approach was implemented by condensing reverse-engineered class diagrams of software systems into compact ones. In Ref [53], McBurney et al. built several ANN (Artificial Neural Network) based prediction models to classify classes as either *important* or *unimportant*. They believed that these important classes should be documented first.

We built KCP models using different metric suites (i.e., DMs, NMs_*u*_, NMs_*w*_, and their combinations) and two machine-learning algorithms—Random Forest and NaiveBayes. Thus, this study can be grouped into the category of *supervised approaches*. Note that while KCP has seen notable progress, several key challenges remain unresolved (cf. Sect 1 for details). Furthermore, different from the existing studies that focused on improving the performance of KCP models, our study is performed from a different angle, that is, we aim to thoroughly investigate the effectiveness of network metrics on KCP.

Note that previous studies revealed that DMs+NMs performed better than DMs on KCP. However, they did not examine whether NMs alone are better than DMs. At the same time, the class coupling networks they built are not very accurate, ignoring many nodes (i.e., classes), many edges (i.e., couplings), and also the weights on edges, which leads to questionable metric values and unreliable conclusions. In this study, we build a more accurate class coupling network—${\text{CCN}}_{\text{WD}}$. Thus, our metric values are more accurate, and the obtained conclusions are more reliable. We find that DMs+NMs does perform better than using DMs alone, which is consistent with previous work. At the same time, ${\text{CCN}}_{\text{WD}}$ and weighted network metrics allow us to comprehensively investigate the relative effectiveness of network metrics over DMs. Specifically, we also reveal the relative effectiveness of NMs_*w*_, DMs+NMs_*w*_, NMs+NMs_*w*_, and DMs+NMs+NMs_*w*_ over DMs (cf. Sect 3.1 for details).

Furthermore, to our knowledge, previous studies have never explored KCP in the cross-project context. In this sense, our work fills the gap. We obtain many interesting results, which have not been found in the within-project context. For example, we find that in the cross-project context, DMs performs better than NMs on KCP, which contradicts the conclusion obtained in the within-project context. Such an observation suggests that network metrics do not always perform better than DMs. In the cross-project context, we should choose DMs to build KCP models (cf. Sect 3.2 for details).

### 6.2 Unsupervised approaches

Ref [7] coined the *key classes* concept and proposed an approach to identify key classes. Their approach is based on a graph representation of a system through dynamic analysis and the HITS (Hypertext-Induced Topic Search) webmining algorithm to compute class importance. Ref [11] built graphs to represent software systems through static analysis and employed the PageRank algorithm to identify possible key classes. Ref [12] represented software as dependency graphs through static analysis, and employed both *a*–*index and h*-index to identify possible key classes. Ref [54] represented software as dependency graphs through static analysis, and utilized some network metrics to compute class importance so as to identify possible key classes. In Ref [13], Meyer et al. represented software as unweighted undirected software networks through static analysis, and employed *k*-core decomposition to identify possible key classes. In Ref [14], Pan et al. represented software as weighted directed software networks through static analysis, and utilized a generalized *k*-core decomposition to identify possible key classes. Ref [55] and [15] proposed a recommender system to identify possible key classes in software systems. Their system used PageRank algorithm and its variants to compute class importance. Vale and Maia [56] proposed a dynamic-analysis-based approach, Keecle, to identify possible key classes. In Ref [8], Pan et al. treated software as multilayer networks, and employed both AHP (Analytic Hierarchy Process) and a new PageRank variant to identify key class candidates. In Ref [17], Pan et al. proposed a Pride approach to identify key classes so as to prioritize code documentation effort. Pride used WDCCNs (the same as the ${\text{CCN}}_{\text{WD}}$s used in this work) to represent the topological structure of software, and utilized a ClassRank algorithm to quantify class importance. In Ref [19], Pan et al. proposed an iFit approach to identify possible key classes. Their approach is inspired by the *field theory* in physics and thus can consider the impact of both contact and non-contact couplings between classes on the class importance.

## 7 Conclusions and future work

In this work, we investigate the effectiveness of network metrics on KCP across two contexts (i.e., within-project and cross-project) through an empirical study on 18 open-source Java projects. In the within-project KCP context, our results suggest that i) network metrics (i.e., NMs or NMs_*w*_) alone or along with design metrics (i.e., DMs) are superior to DMs alone; ii) weighted network metrics (NMs_*w*_) perform better than both NMs and NMs+DMs; and iii) the above observations hold for all the three considered weighting mechanisms. In the cross-project KCP context, our results suggest that i) design metrics (i.e., DMs) perform better than network metrics (i.e., NMs or NMs_*w*_); ii) network metrics (i.e., NMs or NMs_*w*_) along with design metrics outperform network metrics (i.e., NMs or NMs_*w*_) alone; and iii) weighting mechanisms have slight impact on the relative effectiveness of different metric suites on KCP.

Note that though we collected the largest set of subject systems in the KCP literature for our experiments, we cannot ensure the generalization of the obtained results. But at least we can observe that there exist software projects where the above conclusions hold true. Thus, we suggest that when building KCP models, researchers and practitioners should consider using network metrics (i.e., NMs or NMs_*w*_) alone or along with design metrics in the within-project KCP context, using design metrics alone or along with network metrics (i.e., NMs or NMs_*w*_) in the cross-project KCP context, and using network metrics (i.e., NMs or NMs_*w*_) along with design metrics across KCP contexts.

Our future work includes: i) exploring the effectiveness of other OO metrics on KCP, ii) exploring the impact of different classification techniques on KCP, and iii) investigating the impact of network metrics on more non-Java/closed-source software systems.
